# Alcohol Use as Risk Factors for Older Adults’ Emergency Department Visits: A Latent Class Analysis

**DOI:** 10.5811/westjem.2015.9.27704

**Published:** 2015-12-08

**Authors:** Namkee G. Choi, C. Nate Nathan Marti, Diana M. DiNitto, Bryan Y. Choi

**Affiliations:** * The University of Texas at Austin, School of Social Work, Austin, Texas; † Brown University, Department of Emergency Medicine, Providence, Rhode Island

## Abstract

**Introduction:**

Late middle-aged and older adults’ share of emergency department (ED) visits is increasing more than other age groups. ED visits by individuals with substance-related problems are also increasing. This paper was intended to identify subgroups of individuals aged 50+ by their risk for ED visits by examining their health/mental health status and alcohol use patterns.

**Methods:**

Data came from the 2013 National Health Interview Survey’s Sample Adult file (n=15,713). Following descriptive analysis of sample characteristics by alcohol use patterns, latent class analysis (LCA) modeling was fit using alcohol use pattern (lifetime abstainers, ex-drinkers, current infrequent/light/moderate drinkers, and current heavy drinkers), chronic health and mental health status, and past-year ED visits as indicators.

**Results:**

LCA identified a four-class model. All members of Class 1 (35% of the sample; lowest-risk group) were infrequent/light/moderate drinkers and exhibited the lowest probabilities of chronic health/mental health problems; Class 2 (21%; low-risk group) consisted entirely of lifetime abstainers and, despite being the oldest group, exhibited low probabilities of health/mental health problems; Class 3 (37%; moderate-risk group) was evenly divided between ex-drinkers and heavy drinkers; and Class 4 (7%; high-risk group) included all four groups of drinkers but more ex-drinkers. In addition, Class 4 had the highest probabilities of chronic health/mental problems, unhealthy behaviors, and repeat ED visits, with the highest proportion of Blacks and the lowest proportions of college graduates and employed persons, indicating significant roles of these risk factors.

**Conclusion:**

Alcohol nonuse/use (and quantity of use) and chronic health conditions are significant contributors to varying levels of ED visit risk. Clinicians need to help heavy-drinking older adults reduce unhealthy alcohol consumption and help both heavy drinkers and ex-drinkers improve chronic illnesses self-management.

## INTRODUCTION

Older adults (65+ years of age) consistently account for the largest proportion of emergency department (ED) visits/repeat visits, and they are expected to become an even larger presence in the ED when the “baby boomers” swell the ranks of older adults.^1^–^4^ Between 2006 and 2011, older adults’ ED visits increased by 2.3%, and visits by the 45–64 age group increased by 8.3%,^5^ signaling a steep increase in visits by older adults in the coming years. Data also show that ED visits by individuals with substance use disorders have been increasing (a 34% increase for alcohol-related disorders and 48% increase for other substance-related disorders between 2006 and 2011).^5^ Given that the boomers have had higher rates of substance use/misuse than their predecessors,^6^,^7^ the growing numbers of older adults who use/misuse substances are likely to crowd EDs, requiring the examination of substance use/misuse’s impacts on ED visits by late middle-aged and older adults. Using nationally representative data on individual health status and healthcare utilization, this study sought to identify subgroups of individuals aged 50+ for their ED visit risk based on alcohol use patterns, chronic health and mental health conditions, and previous ED visits.

People aged 50+ have lower rates of heavy alcohol use and alcohol abuse/dependence than younger adults, because both alcohol use and drinking quantity tend to decline with age and increasing chronic disease burden.^8^–^13^ However, even low-to-moderate alcohol use in late life can predispose older adults to adverse health outcomes, as aging- and disease-related physiological changes (e.g., smaller body mass and lower total body water content) lead to higher and longer-lasting blood alcohol content and neurotoxicity in older than in younger adults.^14^,^15^ Despite general findings of the beneficial health effects of low-to-moderate drinking, the overall net effect of alcohol consumption on health outcomes is detrimental, owing to the negative effect on cancers; infectious disease; cardiovascular, hepatic, endocrine, and gastrointestinal diseases; neuropsychiatric disease including alcohol-use disorders; and intentional and unintentional injuries.^16^–^19^

Epidemiologic data from the 2008–2012 National Survey on Drug Use and Health (NSDUH) showed that 11% of the 50–64 age group and 20% of the 65+ age group were lifetime abstainers, and 21% of the 50–64 age group and 28% of the 65+ age group were ex-drinkers (i.e., did not use alcohol in the preceding 12 months).^20^ Compared to lifetime abstainers and current drinkers, ex-drinkers have been found to have more physical and mental health problems and are likely to include “sick quitters” who stopped drinking heavily due to health problems that are caused by or deteriorated because of long-term alcohol use.^21^,^22^

In reality, a substantial proportion of those aged 50+, with or without chronic medical conditions, continue to engage in at-risk/harmful/hazardous drinking. The 2013 NSDUH show that 23% of the 50–54 years old, 16% of the 55–59 years old, 14% of the 60–64 years old, and 9% of the 65+ years old were binge (but not heavy) alcohol users (i.e., defined as 5+ drinks on the same occasion on at least 1 day in the past 30 days ); and 6%, 4%, 5%, and 2% in each respective group were heavy users (5+ drinks on the same occasion on each of 5 or more days in the past 30 days).^8^ A study based on the 2005–2007 NSDUH data also found that among alcohol users, 20% of those aged 50–64 and 15% of those aged 65+ endorsed alcohol abuse or dependence symptoms.^23^ Another study, based on the 2005–2008 National Health and Nutrition Examination Survey data and using the alcohol-related risk assessment algorithm, also found that in the context of their medical problems, functional status, and other health risks, 37% of drinkers aged 65+ were classified as engaging in harmful consumption (based on both frequency and amount of alcohol intake), and 53% engaged in either harmful or hazardous consumption.^24^ The study also found that male drinkers and Black drinkers had significantly greater odds of hazardous/harmful consumption than female and White drinkers.^24^

Other studies based on Medicare beneficiaries or primary care patients corroborate these epidemiologic findings. That is, 31% of community-dwelling, fee-for-service Medicare beneficiaries aged 65+ with at least one of seven chronic conditions (i.e., Alzheimer’s disease or other dementia, chronic obstructive pulmonary disease, depression, diabetes, heart failure, stroke, and hypertension) reported alcohol consumption, and 7% reported at-risk drinking (i.e., 30+ drinks per typical month or 4+ drinks in any single day).^13^ Nearly 35% of current drinkers aged 60+ seen at primary care settings engaged in at-risk drinking behaviors that included any of the following: (1) alcohol use despite high-risk comorbidities (e.g., liver disease, pancreatitis, high blood pressure, gout, heartburn, stomach pain, falling, nausea, memory impairment, depression); (2) alcohol use despite high-risk medication use (medications that may cause bleeding, dizziness, sedation and those for hypertension, ulcer disease, gastroesophageal reflux, and depression); and (3) at-risk alcohol use alone (e.g., binge drinking, driving under the influence).^25^

Older adults who misuse alcohol have higher rates of ED visits than their age peers who do not misuse alcohol. The National Institute on Alcohol and Alcohol Abuse and American Geriatrics Society guidelines use lower guidelines than the NSDUH for defining heavy drinking among older adults, i.e., 4+ drinks in any single day during a typical month in the past year. Compared to their age peers who drink within these guidelines, older-adult heavy drinkers had a 1.91 greater odds (95% CI [1.11–3.30]) of acute care ED utilization for ambulatory-care sensitive conditions.^26^ Regardless of age group, repeat ED users were also found to include a higher proportion of those with alcohol-related diagnoses than non-repeat users.^27^,^28^

Higher ED visit rates among older adults who misuse alcohol are attributable in part to alcohol’s adverse effects on chronic medical conditions, falls and other accidents resulting in fractures, self-inflicted injuries including suicide attempts, delirium, gastrointestinal problems, alcohol/alcohol-withdrawal induced mood disorders and agitation, lower adherence to prescribed therapy for chronic medical conditions, and lower rates of primary care and preventive care visits.^4^,^26^, ^29^–^35^ Older-adult alcohol and/or drug users who take multiple prescription and nonprescription medications are also at a high risk for potentially dangerous interaction effects between these medications and substance use.^36^,^37^ Those who concurrently use alcohol with opioid pain relievers (OPR) or benzodiazepines (BZD) are at an especially high risk for fatal/nonfatal overdose, more aberrant behaviors, accidents, and greater ED visits.^38^,^39^ The 2010 Drug Abuse Warning Network data showed that alcohol was involved in nearly 13% of OPR abuse-related ED visits and nearly 25% of BZD abuse-related visits among patients aged 55+.^40^

ED visits have negative health and mental health consequences for older adults.^41^,^42^ A systematic review found that between one-third and one-half of ED patients aged 65+ are admitted to a hospital, which is 2.5–4.6 times higher than the hospital admission rates among younger ED patients.^31^ One study also found that problem drinking was associated with worse self-perceived health among older patients in the year following an ED visit.^43^ Frequent ED visits by increasing numbers of older adults are also likely to further increase healthcare costs and drain healthcare resources.^44^ Since alcohol-related health crises can be prevented, identification of subgroups of late middle-aged and older adults who may be at a high risk of ED visits and frequent visits based on their health status and alcohol use/misuse patterns is important for helping older adults avoid such visits.

In this study, we used latent class analysis (LCA)^45^,^46^ to identify unobservable subgroups of individuals aged 50+ who may be at risk of ED visits based on their alcohol nonuse/use patterns, chronic health and mental health conditions, and previous ED use. The study contributes to the ED literature by examining ED visit risk levels incorporating health status and alcohol consumption patterns among the population group that comprises the largest share of ED users.

## METHODS

### Data Source and Sample

Data came from the 2013 National Health Interview Survey (NHIS). The annual, cross-sectional NHIS series is the principal source of information on the health of the civilian noninstitutionalized population of the United States.^47^ The 2013 NHIS public-use data file contains information on 41,336 households and 42,321 families, with 12,860 children and 33,557 adults interviewed as sample children and sample adults, respectively. All interviews were done face-to-face. Of the total 16,505 sample adults aged 50 years and older, the present study focused on 15,713 respondents, after excluding 619 (4.25%) who were not self-interviewed (i.e., proxy interviewed or interviewee status not known) and an additional 173 (1.01%) whose alcohol-use data were missing.

### Measures: Latent Class Indicators

Alcohol nonuse/use pattern was categorized into lifetime abstainers, ex-drinkers, current infrequent/light drinkers, current moderate drinkers, and current heavy drinkers. The NHIS defines lifetime abstainers as those who have had less than 12 drinks of any alcoholic beverages (including liquor such as whiskey or gin, beer, wine, wine coolers, or any other type of alcoholic beverages) in their entire life. Ex-drinkers had had 12+ drinks in their lifetime but had not consumed any alcoholic beverages in the past year. Current drinkers had had 12+ drinks in their lifetime and at least one drink in the past year. Based on the frequency and number of drinks in the past year, current infrequent drinkers had 1–11 drinks total; current light drinkers had 3 or fewer drinks per week; current moderate drinkers had 4–14 drinks per week for men and 4–7 drinks per week for women; and current heavy drinkers had 15+ drinks per week for men or 8+ drinks per week for women.^47^ Since our bivariate and multivariate analyses showed no significant difference in the numbers of diagnosed chronic illnesses and other reports of chronic health conditions among current infrequent, light, and moderate drinkers, we combined these three groups in the LCA in this study.

Chronic health and mental health conditions (yes=1; no=0 for each) included: (1) chronic illnesses (hypertension [HP], heart disease [coronary heart disease, angina pectoris, myocardial infarction, and/or other health disease or condition], stroke, diabetes, any lung problems [asthma, chronic obstructive pulmonary disease-COPD, emphysema], arthritis [arthritis, rheumatoid arthritis, gout, lupus, fibromyalgia], and cancer as diagnosed by a doctor or other health professional); (2) chronic (in the past three months) fracture, bone/joint injuries that caused functional limitations; (3) chronic (in the past three months) depression/anxiety/other emotional problems that caused functional limitations; (3) chronic (in the past three months) experience of pain in neck, low back, face/jaw muscles and joints, head/migraine, and generalized joint pain that lasted a whole day or more; and (4) whether or not the respondent needed help with activities and instrumental activities of daily living (ADL/IADL).

Number of ED visits in the past 12 months was measured with the question, “…how many times have you gone to a hospital emergency room about your own health (this includes emergency room visits that resulted in a hospital admission)?” The response categories were 0, 1, 2–3, 4–5, 6–7, 8–9, 10–12, 13–15, 16 or more.”

### Measures: Sample and Latent Class Membership Characteristics

Sample and latent class membership characteristics included demographics, self-rated health and mental health status, health-related behaviors, and healthcare service use (in the past 12 months).

Demographics were chronological age and age group (50–59, 60–69, 70–79, & 80+ years); gender (male vs. female); race/ethnicity (non-Hispanic white, non-Hispanic Black, Hispanic, non-Hispanic Asian, other); marital status (married/cohabiting vs. not married/cohabiting); education (college degree vs. no college degree); employment status (employed vs. not employed); and region of residence (Northeast, Midwest, South, and West).

Self-rated health was measured on a 5-point scale (1=poor, 5=excellent); and mental health status was measured with the six-item K6 for psychological distress (“feeling nervous; feeling hopeless; feeling restless or fidgety; feeling so sad or depressed that nothing could cheer you up; feeling that everything was an effort; and feeling down on yourself, no good, or worthless” during the past 30 days; 0=none of the time, 4=all of the time).^48^ Cronbach’s alpha for the study sample was .88. Due to individual item missing values, K6 scores were grouped into no symptoms (=0), any symptoms (≥1), and missing.

Health-related behaviors included (1) body mass index (BMI) calculated from the respondent-reported height and weight, without shoes, at the time of the survey (underweight, healthy weight, overweight, obese, missing); (2) leisure time physical activities (exercise, sports, physically active hobbies…) referring to engagement at least once a week in vigorous, low/moderate, or strength activities; and (3) any tobacco product use (current daily or some-day user, former user, never user) including cigarette smoking and/or other tobacco product use.

Healthcare service utilization in the past 12 months (yes=1; no=0 for each) included (1) insurance status (private insurance, Medicare, and Medicaid); (2) visit with a general doctor/primary care physician (general practice, family medicine, or internal medicine); (3) visit with a mental health service provider (psychiatrist, psychologist, psychiatric nurse, or clinical social worker); and (4) whether or not the ED was the respondent’s usual source of healthcare.

### Data Analytic Approach

LCA is a method for identifying unobserved subgroups (latent classes) that consist of individuals that share similar characteristics across a variety of measures.^45^,^46^ In this study, we used LCA to identify latent subgroups of older adults based on alcohol consumption patterns, chronic physical and mental health conditions, and past-year ED visits. The LCA models were fit using Mplus 7.13^49^ using full information maximum likelihood estimation with robust standard errors, which makes use of all available data.^50^ The first step in fitting an LCA model is to determine the optimal number of classes that underlie the population. This was done by fitting a series of models beginning with a one-class model in which all respondent were treated as a single population, then sequentially increasing the number of classes until there was no improvement gained by adding an additional class. Simulation studies that examined the properties of fit indices^51^,^52^ and null hypothesis significance tests^52^ concluded that the Bayesian information criterion (BIC) and the sample-adjusted BIC were the best indicators of class recovery; however, the Lo-Mendell-Rubin (LMR) likelihood ratio test^53^ and the bootstrap likelihood ratio test performed similarly well. In another LCA simulation study, Clark and Muthén^54^ demonstrated that true parameter values are more likely to be in the 95% confidence when entropy, a measure of classification accuracy, was greater than .80. For this study, a series of models were fit and evaluated using LMR likelihood ratio test; entropy (>0.80)^54^; average class probabilities (>0.80)^55^; a scree plot of the BIC; and inspection of latent classes’ descriptive statistics. After fitting the LCA model, LCA membership was used as an independent variable in a series of generalized linear models using an identity link function for continuous outcomes and a logit link function for binary outcomes to assess class differences in demographic and other characteristics. All estimates presented in this study are weighted, with the exception of sample sizes.

## RESULTS

### Sample Characteristics by Alcohol Nonuse/Use Patterns

As alcohol consumption pattern was one of the key indicators for LCA, data in Tables 1 and 2 describe the characteristics of the five alcohol nonuse/use groups. Table 1 shows sociodemographic and health behavior characteristics of the study sample by alcohol nonuse/use pattern. It shows that 20% were lifetime abstainers, 20% were ex-drinkers, 26% were current infrequent/light drinkers, 14% were current moderate drinkers, and 19% were current heavy drinkers. Lifetime abstainers and ex-drinkers were older than the three current drinker groups; however, of all five groups, lifetime abstainers had the highest proportion of women, racial/ethnic minorities, and never smokers.

Table 2 shows health status and healthcare use characteristics by alcohol nonuse/use pattern. Ex-drinkers had the poorest health and mental health indicators. A significantly higher proportion of lifetime abstainers (the oldest of the five groups) than current infrequent/light drinkers and current moderate drinkers also had chronic illnesses and needed help with ADL/IADL, but they were least likely of all five groups to report any psychological distress symptoms. Current infrequent/light drinkers and current moderate drinkers were similar to each other in health and mental health indicators and had the fewest chronic illnesses of all groups. Compared to these two groups of current drinkers, a larger proportion of current heavy drinkers had reported chronic illnesses, needed help with ADLs/IADLs, and reported psychological distress symptoms. Heavy drinkers were also mostly likely (8.17%) to have reported functional limitations due to chronic bone/joint fractures or other injuries.

With respect to past-year healthcare use, 77% of lifetime abstainers and about 80% of the other groups visited a general doctor/primary care physician. Lifetime abstainers had the smallest portion of mental health service users. Almost 25% of ex-drinkers, about 20% of lifetime abstainers and current heavy drinkers, and about 16% of current infrequent/light drinkers and current moderate drinkers visited an ED in the past 12 months.

### Determination of Number and Interpretation of Latent Classes

Fit indices, entropy, LMR, and average class probabilities for models with two through five classes are presented in Table 3. After evaluating all five LCA models (i.e., models with 1 through 5 latent classes), a four-class model was selected for subsequent analyses as the LMR test indicated no significant difference between the 4-class and 5-class models which indicates that the additional complexity of a 5-class model relative to a 4-class model did not improve the model fit; other indices did not appreciably differ across models. The Figure shows item proportions for each of the four latent classes (i.e., the proportions of individuals in a putative class that possesses given characteristics). On the basis of the class characteristics in the Figure, classes were characterized as follows: Class 1: lowest risk, Class 2: low risk, Class 3: moderate risk, and Class 4: high risk.

Table 4 shows model parameters for the four classes (Class 1: 35% of the sample, Class 2: 21%; Class 3: 37%; and Class 4: 7%) in the probability scale and the ascending order of ED visit risks from Class 1 to Class 4. All pairwise comparisons between classes were either non-estimable (NE) due to perfect prediction or were significantly different at p<0.001. Class 1 members were almost exclusively infrequent/light/moderate drinkers (i.e., probability >0.99); Class 2 members were almost exclusively lifetime abstainers (i.e., probability >0.99); and Class 3 members were evenly divided between ex-drinkers (probability =0.505) and heavy drinkers (probability =0.495). Class 4 members included all four drinking groups but more heavily ex-drinkers (probabilities =0.333 for ex-drinkers; 0.275 for infrequent/light/moderate drinkers; 0.212 for lifetime abstainers; and 0.179 for heavy drinkers) and were also almost exclusively those who had repeat ED visits in the past year. As expected, Class 1 had the lowest probability and Class 4 had the highest probability of all chronic illnesses, chronic mental health problems, chronic pain, need for ADL/IADL help.

In sum, Class 3 members were ex-drinkers and heavy drinkers who also had higher probabilities of chronic disease burden than Classes 1 and 2 members. Class 4 members also included higher proportions of ex-drinkers and heavy drinkers than Classes 1 and 2 members. In addition, compared to the other three classes, Class 4 members had the highest probabilities of all chronic physical, functional, and mental health conditions and a history of repeat ED visits.

### Latent Class Membership Characteristics

Table 5 shows that relative to the other three classes, Class 1 members (lowest risk group) were younger and included higher proportions of men, non-Hispanic Whites, married/cohabiting persons, college graduates, employed persons, Northeast residents, those with excellent self-rated health, those doing weekly physical activities, and those with private insurance. Class 1 members also included a higher proportion of overweight people, but a lower proportion of obese people. Relative to the other three classes, Class 2 members (low-risk group) were older and included higher proportions of women, Hispanics, Asians, Southerners, those with no psychological distress symptoms, and those who never used tobacco products. Relative to the other three classes, Class 3 members (moderate risk group) included a highest proportion of Midwesterners. Relative to the other three classes, Class 4 members (high-risk group) included higher proportions of non-Hispanic Blacks (20%), not married/cohabiting persons (49%), obese persons (39%), current smokers (25%), and Medicare- (53%) and Medicaid- (18%) covered persons and lower proportions of college graduates (16%) and those who did any weekly physical activity (47%). Class 4 members were most likely to have visited a general doctor (90%) and to report the ED as their usual healthcare source when sick (3.2%).

## DISCUSSION

This study identified four classes of individuals aged 50+ with regard to their ED visit risk levels by examining their alcohol consumption patterns and health status. The findings show that alcohol consumption patterns are a significant indicator for ED visit risk, with infrequent/light/moderate drinkers and lifetime abstainers presenting significantly lower risk probabilities than ex-drinkers and heavy drinkers. As expected, in addition to being infrequent/light/moderate drinkers, Class 1, the lowest-risk group, is also the youngest and the healthiest by all indicators.

In contrast to Class 1, Class 2 members were the oldest of all four classes and had significantly lower socioeconomic status (SES; i.e., more racial/ethnic minorities and fewer college graduates and employed persons). Class 2 was exclusively lifetime abstainers, and despite their older age and low SES had lower rates of chronic health conditions, functional limitations, and mental health problems than Classes 3 and 4. Previous studies show that lifetime abstainers are often genetically predisposed to or have chosen abstention because of their religious beliefs, culture, or family environment and personal values and beliefs about alcohol or other substance use and can thus avoid substance-induced/influenced risky behaviors.^56^–^58^ Other recent studies also show that lifetime abstainers tend to have a more favorable cardiovascular profile and better overall mental health than ex-drinkers or heavy or binge drinkers.^37^,^59^

The high probability of ex-drinkers in Classes 3 and 4, the two higher risk groups, with significantly poorer health/mental health than current infrequent/light/moderate drinkers and lifetime abstainers, supports the “sick quitter” assumption and the possibility that ex-drinkers were likely to include former bingers and heavy drinkers. For example, Ng Fat et al.^60^ found that worsening health or preexisting poor health and poor psychosocial health were associated with ceasing alcohol consumption at ages 42 and 50. Class 4 members have the highest burden of chronic diseases (especially cardiovascular diseases), chronic pain, and chronic mental health problems. Nevertheless, a substantial proportion of them engage in unhealthy behaviors as shown in their rates of heavy drinking, obesity, smoking, and no physical activity. Although older adults’ medical conditions causing chronic pain tend to reduce alcohol consumption over time, some rely on alcohol to manage pain, which leads to more alcohol consumption and/or alcohol-related problems.^61^–^63^

Unhealthy behaviors among Class 4 members may also stem from their significant SES disadvantages, which may not facilitate adoption of healthy behaviors and effective self-management of chronic medical conditions. Although a majority of Class 4 members appear to have a usual place of healthcare other than the ED, ED visits were likely for health crises resulting from high disease burden and unhealthy behaviors even for those with primary care access.

The findings have the following clinical and research implications. First, primary care physicians and other aging-service providers should provide their patients at high risk for ED visits with more psychoeducation or other such interventions that will encourage reducing problematic alcohol consumption and engaging in other healthy behaviors. Almost all interventions for treatment-seeking older-adult substance abusers demonstrate positive outcomes that are on par with those among younger cohorts.^64^ Brief advice or brief interventions at primary-care settings for non-treatment-seeking older-adults with alcohol-related problems have also had positive effects; however, long-term effects of these brief interventions have been mixed.^64^ More research on longer-term, age-specific interventions is needed.

Second, all older adults with chronic illnesses are likely to benefit from better self-management of chronic illnesses by participating in evidence-based programs such as Stanford’s Chronic Disease Self-Management Program, which has been found to reduce ED visits and hospitalizations among participants.^65^ Primary care and ED physicians should refer their patients to face-to-face or web-based chronic disease self-management programs.

Third, access to preventive care in primary care settings and mental health services needs to be improved for those at high risk of ED visits. Especially given the high-risk group’s low SES, transportation and other barriers to accessing primary care should be examined.

Fourth, given the mixed evidence about brief interventions in EDs, trauma care centers, and in-patient hospital care settings with regard to their effects on treatment and healthcare utilization outcomes, more research is needed to refine treatment practice and enhance treatment outcomes.^66^,^67^

Finally, in addition to individual-level interventions, efforts to improve preventive healthcare access and healthy behaviors require mezzo- and macro-level interventions such as neighborhood-level public health interventions and higher Medicaid and health insurance reimbursement for preventive services.

## LIMITATIONS

Our study has some limitations due to data constraints. First, data on ED visits did not include the circumstances leading to these visits, which would have provided a richer contextual description. Second, since ED discharge diagnosis was also missing, potential interaction effects of alcohol, prescription drugs, and over-the-counter medications and alcohol’s effects on falls and other injuries on ED visit risk could not be factored in. Third, the LCA indicators, including past-year ED visits, were self-reported and possibly subject to underreporting due to poor recall or social desirability bias. Fourth, NHIS does not collect data on illicit drug use/misuse. Given the continuing increase in drug-use disorders among ED visitors,^5^ future research should include both alcohol and drug use/misuse.

## CONCLUSION

Alcohol nonuse/use and quantity of use contribute significantly to varying levels of ED visit risk among individuals aged 50+. In the face of projected increases in ED visits by this age group, the findings underscore the importance of clinical practice that takes into account past and current alcohol use/misuse and provides psychoeducation and other interventions to increase healthy behaviors. In particular, clinicians need to help heavy-drinking older adults reducing unhealthy alcohol consumption and help both heavy drinkers and ex-drinkers improve chronic illnesses self-management. More research on effective treatment practices in primary care and other healthcare settings for older adults with alcohol-related problems is needed. On mezzo- and macro-levels, improving access to preventive care in primary care setting and mental health treatment, especially for older adults with low SES and high chronic disease burden and poor mental health, is a necessity.

## Figures and Tables

**Figure:**
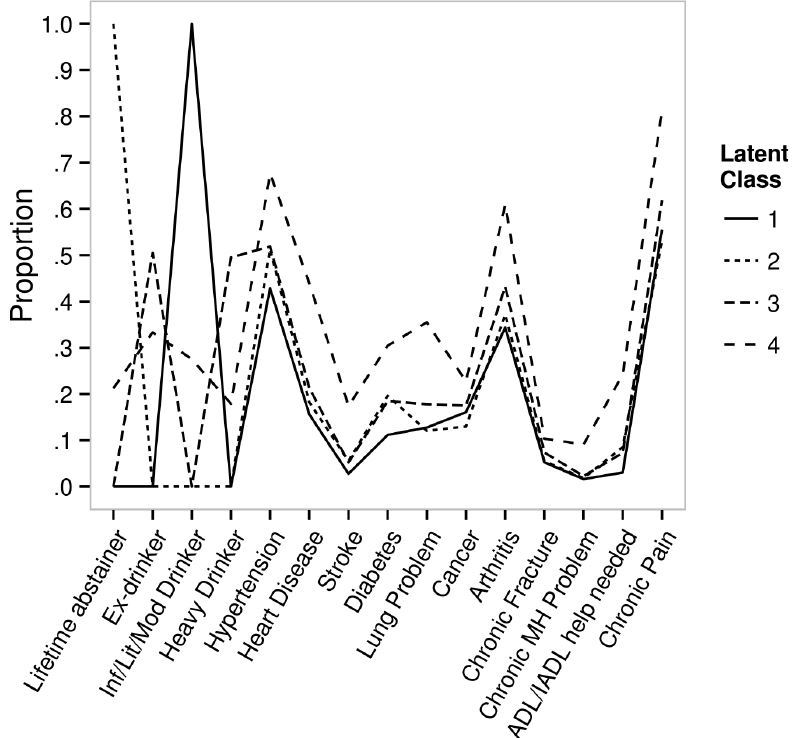
Indicators across latent classes. The figure presents the proportions of respondents within each class that exhibit each of the characteristics that were used to determine the latent classes (e.g., 100% of Class 1 is comprised of infrequent/light/moderate drinkers, slightly over 40% of Class 1 exhibits hypertension, etc.). *ADL,* activities of daily living; *IADL,* instrumental activities of daily living

**Table 1:** Sociodemographic characteristics and health-related behaviors by alcohol nonuse/use pattern.

N (%)	All 15,713 (100)	Lifetime abstainer 3,505 (20.02)	Ex-drinker 3,474 (20.37)	Current infrequent/light drinker 3,759 (26.18)	Current moderate drinker 2,069 (14.37)	Current heavy drinker 2,906 (19.05)
Sociodemographics						
Chronological age (M,SE)	63.58 (0.11)	66.40^a^ (0.25)	65.50^b^ (0.22)	61.32^c^ (0.19)	61.97^d^ (0.24)	62.89^e^ (0.24)
Age group (%)						
50–59 years	41.49	32.72	33.97	49.27	47.93	43.21
60–69 years	31.51	28.99	32.40	32.13	31.22	32.56
70–79 years	17.58	22.69	20.34	13.41	15.14	16.83
80+ years	9.42	15.60	13.29	5.20	5.71	7.40
Male (%)	46.70	28.47	50.22	49.76	69.12	40.95
Race/ethnicity (%)						
Non-Hispanic White	75.45	61.74	72.88	79.79	84.94	79.51
Non-Hispanic Black	10.47	14.37	13.32	8.34	6.09	9.54
Hispanic	9.20	14.49	9.48	7.93	6.15	7.37
Non-Hispanic Asian	4.20	8.55	3.10	3.47	2.65	2.97
Other	0.68	0.85	1.23	0.47	0.17	0.61
Married/cohabiting (%)	64.33	59.00	58.61	69.89	72.21	62.46
College degree (%)	30.69	22.22	19.48	40.19	43.98	28.48
Employed (%)	51.16	40.60	38.42	62.09	61.37	53.14
Health behaviors						
Body mass index (%)						
<18.5 (underweight)	1.41	1.84	1.71	1.11	1.16	1.23
18.5–24.99 (healthy)	29.91	31.18	27.31	29.31	33.50	29.48
25–29.99 (overweight)	36.19	33.53	33.96	37.69	42.01	34.92
30+ (obese)	29.44	28.91	34.62	29.33	21.55	30.55
Missing	3.05	4.53	2.40	2.56	1.79	3.82
Any type of leisure time physical activity at least once a week (%)	63.34	49.12	55.84	71.48	76.04	65.53
Vigorous activity	32.09	21.05	23.55	40.79	44.24	31.71
Moderate/light activity	54.34	40.43	48.43	61.38	65.61	57.12
Strengthening activity	21.71	12.61	15.70	27.79	30.59	22.63
Tobacco use (%)						
Current user	18.84	8.89	20.02	18.30	25.15	24.02
Former user	36.14	16.78	43.65	40.44	44.01	36.62
Never user	45.02	74.33	36.34	41.26	30.84	39.35

All group differences are significant at p<0.001.

*M,* mean; *SE,* standard error of the mean

a–e F(4,297)=99.21 for chronological age (Bonferroni-corrected): c=d<b<a; d=e; e<a<b<c.

**Table 2:** Health status and healthcare use by alcohol nonuse/use pattern.

N (%)	All 15,713 (100)	Lifetime abstainer 3,505 (20.02)	Ex-drinker 3,474 (20.37)	Current infrequent/light drinker 3,759 (26.18)	Current moderate drinker 2,069 (14.37)	Current heavy drinker 2,906 (19.05)
Health status						
Self-rated health (M,SE)	3.48 (0.01)	3.32^a^ (0.02)	3.19^b^ (0.03)	3.66^c^ (0.02)	3.80^d^ (0.03)	3.49^e^ (0.03)
No. of diagnosed chronic illnesses (M,SE)	1.65 (0.01)	1.68^a^ (0.03)	2.02^b^ (0.03)	1.43^c^ (0.03)	1.38^d^ (0.03)	1.72^e^ (0.03)
Hypertension (%)	49.24	52.79	55.62	43.42	43.60	50.95
Heart disease (%)	19.94	20.41	27.06	16.60	16.06	19.32
Stroke (%)	5.07	6.53	7.79	3.02	3.29	4.79
Diabetes (%)	16.60	20.93	23.08	13.06	8.27	16.25
Asthma (%)	11.49	10.16	12.90	10,90	10.20	13.15
COPD/emphysema (%)	6.58	5.13	10.84	4.71	4.53	7.62
Arthritis (%)	39.76	38.60	47.24	35.83	34.79	42.14
Cancer (%)	16.43	13.68	17.85	16.05	17.10	17.81
Functional limitations due to chronic fractures of bone/joint/other injury (%)	6.36	5.60	7.38	5.65	4.87	8.17
Chronic depression/anxiety/emotional problems (%)	2.35	2.38	3.21	2.00	1.82	2.28
Chronic pain (%)	58.96	54.70	64.76	56.35	56.87	62.39
Need help with ADL/IADL (%)	6.92	9.94	10.93	3.81	3.17	6.58
Psychological distress (%)						
No symptom	47.82	55.41	43.88	47.47	48.75	43.84
Any symptom	50.45	41.98	54.56	50.97	49.64	54.86
Missing data	1.73	2.61	1.56	1.56	1.62	1.30
Healthcare use in the past 12 months						
Emergency department use (%)	19.04	19.72	24.87	15.41	16.14	19.24
Hospitalization (%)	11.30	12.06	14.83	9.18	8.12	12.04
Saw general doctor (%)	79.04	77.07	80.48	79.38	78.98	79.17
Saw mental health provider (%)	6.50	3.76	8.44	7.31	6.57	6.16

All group differences, except asthma (p=0.007), cancer (p=0.004), and chronic depression/anxiety/emotional problems (p=0.063), are significant at p<0.001.

*M,* mean; *SE,* standard error of the mean; *COPD,* chronic obstructive pulmonary disease; *ADL,* activities of daily living; *IADL,* instrumental activities of daily living

a–e F(4,297)=95.13 for self-rated health (Bonferroni-corrected): b<a<e<c<d. F(4,297)=67.08 for number of diagnosed chronic illnesses (Bonferroni-corrected): c=d<a=e<b.

**Table 3:** Latent class analysis model fit indices, entropy, LMR-LRT, and average class probabilities for models with 2 through 5 classes.

No. of latent classes	BIC	Entropy	LMR-LRT	Minimum class probability
2	235902.0	1.00	307764.1 (p<0.001)	1.00
3	225724.6	0.995	293451.7 (p<0.001)	0.996
4	219578.7	0.997	274874.9 (p<0.001)	0.996
5	211715.4	0.998	265012.6 (p=0.140)	0.996

*BIC,* Bayesian information criterion; *LMR,* Lo-Mendell-Rubin; *LRT,* adjusted likelihood ratio test

**Table 4:** Estimated latent class indicators parameter estimates in probability scale, and contrasts between classes.

Indicators	Class 1 (lowest risk) (0.35; n=5,527)	Class 2 (low risk) (0.21; n=3,242)	Class 3 (moderate risk) (0.37; n=5,802)	Class 4 (high risk) (0.07; n=1,142)
Lifetime abstainer	0.000	>0.99	0.000	0.212
Ex-drinker	0.000	0.000	0.505	0.333
Infrequent/light/moderate drinker	>0.99	0.000	0.000	0.275
Heavy drinker	0.000	0.000	0.495	0.179
Hypertension	0.428	0.513	0.519	0.677
Heart disease	0.156	0.182	0.213	0.440
Stroke	0.028	0.055	0.052	0.174
Diabetes	0.112	0.196	0.185	0.304
Lung problem	0.128	0.120	0.178	0.355
Cancer	0.161	0.130	0.175	0.227
Arthritis	0.344	0.372	0.432	0.610
Chronic fracture	0.052	0.055	0.074	0.103
Chronic depression/anxiety/other emotional problem	0.016	0.018	0.023	0.091
Chronic pain	0.555	0.531	0.619	0.810
ADL/IADL help needed	0.030	0.085	0.073	0.242
ED visit-none	0.881	0.859	0.847	0
ED visit-once	0.116	0.132	0.151	0.003
ED visit- 2–3 times	0	0	0	0.735
ED visit- 4+ times	0	0	0	0.262

All paired contrasts between classes are significant at p<0.001 or non-estimable due to perfect prediction.

Note: Emergency department (ED) visits were treated as a continuous variable in the latent class analysis model. The ED visit indicator proportions shown above were obtained using participant’s most likely class membership for display purposes.

*ADL,* activities of daily living; *IADL,* instrumental activities of daily living

**Table 5:** Between latent class differences in sociodemographic, health status, health behavior, and healthcare-use characteristics.

N	Class 1 (lowest risk) (0.35; n=5527)	Class 2 (low risk) (0.21; n=3242)	Class 3 (moderate risk) (0.37; n=5802)	Class 4 (high risk) (0.07; n=1142)
Sociodemographics				
Chronological age (M,SE)	61.55 (0.16)^a^	66.30 (0.25)^b^	64.24 (0.18)^c^	64.28 (0.41)^d^
Age group (%)				
50–59 years	48.50	32.70	38.53	41.46
60–69 years	32.21	29.40	32.23	29.27
70–79 years	14.06	22.83	18.85	16.32
80+ years	5.23	15.07	10.39	12.94
Male (%)	56.82	28.78	46.02	41.34
Race/ethnicity (%)				
Non-Hispanic White	81.82	61.92	77.05	61.11
Non-Hispanic Black	7.25	14.14	10.53	19.23
Hispanic	7.36	14.30	8.32	10.40
Non-Hispanic Asian	3.22	8.85	3.16	2.36
Other	0.36	0.80	0.94	0.91
Married/cohabiting (%)	71.40	60.19	61.43	49.42
College degree (%)	42.39	23.10	24.61	15.78
Employed (%)	62.58	41.86	47.03	31.84
Region of residence (%)				
Midwest	22.74	18.01	24.15	24.00
South	33.27	47.34	36.20	39.68
West	22.00	19.55	21.72	19.06
Northeast	21.99	15.10	17.93	17.26
Self-rated health (%)				
Poor	2.19	4.85	4.17	21.02
Excellent	25.45	17.82	17.83	7.42
Psychological distress (%)				
No symptom	48.95	57.42	45.37	26.15
Any symptom	49.45	39.94	53.27	71.98
Missing	1.61	2.64	1.36	1.88
Body mass index (%)				
<18.5	1.05	1.85	1.32	2.81
18.5–24.99	30.59	31.59	28.77	27.25
25–29.99	39.57	33.78	34.97	29.45
30+	26.47	28.03	31.73	38.95
Missing	2.32	4.75	3.22	1.55
Any type of leisure time physical activity at least once a week (%)	73.53	49.84	62.21	46.83
Vigorous activity	42.49	21.83	28.97	16.16
Moderate/light activity	63.30	41.00	53.92	40.92
Strengthening activity	28.95	12.66	19.84	14.53
Tobacco product use (%)				
Current user	20.57	8.39	21.31	25.15
Former user	41.39	16.55	40.12	39.17
Never user	38.05	75.05	38.57	35.68
Private insurance (%)	69.72	52.39	57.66	42.05
Medicare (%)	32.03	48.22	44.07	52.70
Medicaid (%)	2.97	8.81	6.23	17.97
Saw/talked with a general doctor (%)	78.75	76.00	79.10	89.79
Saw/talked to a mental health provider (%)	6.50	3.35	6.10	18.45
Emergency department was the place most often went when sick (%)	0.34	0.44	0.53	3.21

All group differences are significant at p<0.001.

*M,* mean; *SE,* standard error of the mean

a–e F (3,298)=100.90 for chronological age (Bonferroni-corrected): a<b,c,d; b>c,d; c=d.
